# Effects of enriched branched-chain amino acid supplementation on sarcopenia

**DOI:** 10.18632/aging.103576

**Published:** 2020-07-26

**Authors:** Chun-Hung Ko, Shin-Jiuan Wu, Shan-Tair Wang, Yin-Fan Chang, Chin-Sung Chang, Ta-Shen Kuan, Hua-Ying Chuang, Chia-Ming Chang, Willy Chou, Chih-Hsing Wu

**Affiliations:** 1 Department of Family Medicine Chi Mei Medical Center, Jia-Li, Tainan, Taiwan; 2 Institute of Gerontology, National Cheng Kung University Medical College, Tainan, Taiwan; 3 Department of Food and Nutrition, Chung Hwa University of Medical Technology, Tainan, Taiwan; 4 Ditmanson Medical Foundation Chia-Yi Christian Hospital, Chia-Yi, Taiwan; 5 Department of Family Medicine, National Cheng Kung University Hospital, College of Medicine, National Cheng Kung University, Tainan, Taiwan; 6 Department of Physical Medicine and Rehabilitation, National Cheng Kung University Hospital, College of Medicine, National Cheng Kung University, Tainan, Taiwan; 7 Department of Neurology, Chi Mei Medical Center, Jia-Li, Tainan, Taiwan; 8 Department of Internal Medicine, National Cheng Kung University Hospital, College of Medicine, National Cheng Kung University, Tainan, Taiwan; 9 Department of Physical Medicine and Rehabilitation Chi Mei Medical Center, Jia-Li, Tainan, Taiwan

**Keywords:** BCAA, nutritional supplements, pre-sarcopenia, sarcopenia

## Abstract

To evaluate the effects of short-term administration of enriched branched-chain amino acids (BCAAs) on subjects with pre-sarcopenia or sarcopenia, our quasi-experimental study enrolled 33 subjects (12 pre-sarcopenia/21 sarcopenia; 6 men/27 women; mean age 66.6 ± 10.3 years) to take one sachet (3.6 g) of enriched BCAA powder twice a day for five weeks followed by a discontinuation period of 12 weeks. We evaluated sarcopenic parameters, including grip strength, 6-meter gait speed, and bioelectrical-impedance-analysis-derived skeletal mass index (SMI), at baseline, 5 weeks, and 17 weeks. We found that both pre-sarcopenic and sarcopenic subjects showed improved SMI, gait speed, and grip strength at 5 weeks. However, all three parameters progressively declined at 17 weeks, especially SMI and grip strength in subjects aged < 65 years and gait speed and grip strength in subjects aged ≥ 65 years. It thus appears that supplementation with enriched BCAAs for 5 weeks correlates with short-term positive effects on sarcopenic parameters but attenuation of those effects following discontinuation for 12 weeks.

## INTRODUCTION

Sarcopenia result from a progressive loss of skeletal muscle and is associated with reduced physical function, falls, fractures, disability, hospitalization, and a poor quality of life [1]. Moreover, sarcopenia increases the mortality rate of the elderly by a factor of up to 2.34 fold [2]. The 2018 European Working Group on Sarcopenia in Older People 2 (EWGSOP2) reported that these adverse muscle changes would accrue throughout a lifetime, not only in elderly adults, but also in younger adults [3]. It is estimated that 30% of people > 60 years old, and that half of people > 80 years old might have sarcopenia and a higher-than-average risk for falling [4]. Of all accidental deaths in the USA, 36.8/100,000 population are attributable to falls [5]. Therefore, the EWGSOP2 encouraged more research on sarcopenia to prevent or delay adverse health outcomes that incur heavy burdens on either individuals or healthcare systems [3].

Exercise positively affects muscle mass and strength [6], but the elderly, and especially those that are physically frail, often find it difficult to exercise. Dietary and nutritional interventions may also increase muscle mass and help maintain physical performance and function [7]. Recent studies have claimed that protein supplementation can help prevent sarcopenia [8–10]. Protein is composed of amino acids that can induce a muscle protein anabolic response conditioned by the availability of branched-chain amino acids (BCAAs) such as leucine, isoleucine, and valine [11]. Delayed amino acid absorption and anabolic resistance are common in the elderly [12]. Low levels of BCAAs are associated with low muscle mass, poor muscle function, and low strength in the community-dwelling elderly [13]. Studies report that supplementary BCAAs, leucine, essential amino acids, whey protein, or vitamin D plus low-intensity resistance training, ranging from 3 days to 12 weeks, stimulate muscle protein synthesis and attenuate sarcopenia, even in the elderly confined to bedrest [13–20]. Compliance with supplementation treatment is influenced by the form of nutrients, personal digestion tolerance to oral supplementation, and dosing frequency, among others [21, 22]. Furthermore, few studies have investigated the changes after those above supplementations were discontinued..

The aim of the present study was to evaluate the short-term effects of enriched supplementation with BCAAs in subjects with pre-sarcopenia or sarcopenia. Here, we hypothesized that the physical performance, muscle strength, and muscle mass of elderly patients would improve after 5 weeks of treatment with enriched BCAAs but would decline after a 12-week discontinuation of treatment.

## RESULTS

### Five- week BCAA intervention

The mean short-form MNA score of 33 participants (6 M/27 F; mean age = 66.6 ± 10.3 years) who underwent enriched BCAA treatment was 12.0 ± 1.5, which indicated normal nutritional status (Table 1). After a 5-week enriched BCAA intervention, skeletal mass index (SMI), gait speed, and grip strength were all improved (Table 1). The changes were consistent in both the sarcopenic subgroup (Supplementary Table 1) and the pre-sarcopenic subgroup (Supplementary Table 2), except for SMI.

**Table 1:** Demographic characteristics, physical status, and performance before and after 5 weeks of enriched BCAA treatment.

**Variable**	**Baseline**	**5 weeks**
Case no. (presarcopenia/sarcopenia)	33 (12/21)	
Sex (M/F)	6 M/27 F	
Age (years)	66.6 ± 10.3	
Education (< 6 years)	21 (63.6%)	
Smoking habit	4 (12.1%)	
Alcohol drinking habit	2 (6.1%)	
Medical history		
Hypertension	10 (30.3%)	
Diabetes	6 (18.2%)	
Dyslipidemia	6 (30.3%)	
Cardiovascular disease	3 (9.1%)	
Thyroid dysfunction	3 (9.1%)	
Height (cm)	154.0 ± 5.6	
Mini Nutrition Assessment Short Form score	12.0 ± 1.5	
International physical assessment activity (kcal/week)	5549.6 ± 4963.1	
Body Weight (kg)	49.7 ± 8.7	50.0 ± 9.2
Body Mass Index (kg/m^2^)	20.9 ± 3.0	21.0 ± 3.2
Body fat (%)	27.8 ± 6.1	27.5 ± 6.6
Waist Circumference (cm)	77.5 ± 12.0	77.9 ± 11.9
Skeletal Muscle Index (kg/m^2^)	5.84 ± 0.91	5.99 ± 1.01*
Gait Speed (m/sec)	0.82 ± 0.17	0.94 ± 0.19**
Grip Strength (kg)	18.0 ± 4.8	21.4 ± 5.1**

Non-parametric Wilcoxon signed rank test: *p < 0.01, **p < 0.001

### Twelve week BCAA discontinuation

No participant dropped out between 0-5 weeks but during the next 12-week discontinuation of BCAAs, there were 7 dropouts. There was no statistical difference in basic characteristics and sarcopenic parameters between the dropouts and the other 26 participants (Supplementary Table 3). The 26 completers in the 5-week intervention group had higher SMI, gait speed, and grip strength than at baseline, but those in the 12-week discontinuation group had lower SMI, gait speed, and grip strength (Table 2).

**Table 2:** Physical status and performance at baseline, after 5 weeks of BCAAs, and after 17 weeks (12 weeks without BCAAs) in 26 completers.

**Variable**	**Baseline**	**5 weeks**	**17 weeks**	**P#**
Case no. (presarcopenia/sarcopenia)		26 (7/19)		
Sex		5 M/21 F		
Age (years)		66.4 ± 10.2		
Height (cm)		154.3 ± 6.0		
Body Weight (kg)	50.4 ± 9.2	50.7 ± 9.9	49.0 ± 9.2	0.832
Body Fat (%)	28.1 ± 6.0	27.9 ± 6.5	28.8 ± 6.2§**	0.004
Skeletal Muscle Index (kg/m^2^)	5.86 ± 0.94	5.99 ± 1.06†	5.80 ± 0.92**	0.006
Gait Speed (m/sec)	0.84 ± 0.16	0.97 ± 0.17*	0.91 ± 0.15§	0.007
Grip Strength (kg)	18.2 ± 4.9	21.7 ± 5.2*	20.9 ± 4.9‡¶	<0.001

#Non-parametric Friedman and Wilcoxon Signed Rank test comparing among baseline, week 5 and week 17: *p < 0.001, †*p* < 0.05 baseline vs. week 5; ‡*p* < 0.01, §*p* < 0.05 baseline vs. week 17; ¶*p* < 0.05, **p < 0.01 week 5 vs. week 17.

### Comparison between BCAA and placebo treatments at five weeks

Before we acquired 33 BCAA interventional samples, our study had 39 subjects at the beginning. However, six of them who agreed to join the intervention study refused to take any BCAA supplements while initiating the intervention. Compared with those six subjects (similar to the open-labeled placebo group), the 12 BCAA interventional subjects (1:2 age and sex matched) showed improved changes in sarcopenic parameters at five weeks (Supplementary Table 4).

### Effect of BCAA on different age groups

To determine the interventional effects on participants in different age groups, participants were divided into two subgroups: those who were < 65 years old and those ≥ 65 years old (Supplementary Figure 1). Grip strength was higher in both subgroups after 5 weeks of BCAA therapy. After 12 weeks of discontinuation, SMI and grip strength were lower than the week 5 test in the < 65-year-old group (1 M/11 F), while grip strength and gait speed were lower than at the week 5 test in the ≥ 65-year-old group (4 M/10 F).

## DISCUSSION

We found that five weeks of supplementation with BCAAs led to increased physical performance, muscle strength, and muscle mass in middle-aged and elderly patients, but the effect subsided after participants discontinued BCAAs for 12 weeks. In the sarcopenic subgroup (n = 21) (Supplementary Table 1), SMI increased, as did grip strength and gait speed (Supplementary Table 1), similar to the results of Takeuchi et al [19]. In the pre-sarcopenic subgroup (n = 12) (Supplementary Table 2), grip strength and gait-speed were higher after BCAA treatment; however, SMI did not change, perhaps owing to our relative small sample size. It is plausible to suggest that a better response to BCAAs can be expected in sarcopenic than in pre-sarcopenic participants.

The duration of BCAA treatment relative to the duration of discontinuation is a critical factor for physical function, grip strength, and muscle mass changes, as demonstrated by results from studies using a wide range of interventional durations, from three days to 12 weeks [13–20]. One study found that muscle protein synthesis (MPS) increased after supplementation with leucine-enriched essential amino acids (containing 3 g of leucine) for just two hours in old women [23]. A recent study using 4.2 g leucine supplementation for six days may also be helpful to increase MPS in the rest leg group of healthy individuals [24]. In agreement with these results, our study here showed an improvement in sarcopenic parameters after five weeks of 7.2 gm BCAAs. On the contrary, several studies have reported that muscle disuse for 10 to 42 days leads to a daily loss of between 0.6% and 5% of muscle mass and to a daily decline in grip strength between 0.3% and 4.2% [17, 25, 26]. We also confirmed lower muscle parameters after a 12-week discontinuation of enriched BCAAs. An interesting finding was the decline of muscle mass followed by a decline in other physical functions and performance. Whether there is also a cause-effect relationship is unclear and warrants further investigation.

Researchers have attempted to determine nutritional strategies to reduce the higher anabolic turnover rate of muscle as humans get older [27]. Leucine is unique because it activates mammalian target of rapamycin complex 1 (mTORC1) and the downstream phosphorylation of p70S6 kinase (p70S6k) and 4E (eIF4E)-binding protein 1 (4E-BP1) and related signaling pathways [28–30]. A 55-mg dose of leucine/kg/d was claimed to be optimal and recommended for younger adults [31] because it activates mTOR and leads to the increased synthesis of muscle protein [32, 33]. Other studies [34, 35] have also reported that leucine supplements helped slow age-related muscle mass reductions in the elderly. Moreover, MPS is stimulated in older mice by leucine-enriched whey protein but not by leucine only [36]. However, one meta-analysis [37] of randomized controlled nutritional interventions for treating sarcopenia reported no effects on appendicular skeletal muscle mass, grip strength, or gait speed. Nevertheless, beta-hydroxy beta-methylbutyrate (HMB), leucine, or BCAAs may strongly promote increases in muscle mass [38, 39]. Compatible with our study here, a randomized controlled trial [40] of eight-week interventions consisting of a leucine-enriched amino acid supplementation (Amino L40; Ajinomoto Co., Inc., Tokyo, Japan) and low-intensity resistance training increased muscle mass, strength, and physical function in post-stroke subjects with sarcopenia. Supplementation with BCAAs shifted the net protein balance from catabolism to anabolism, the result of protein synthesis exceeding protein breakdown [34] and, therefore, an increase in SMI. However, there’s a potential relationship between BCAA supplementation and the development of insulin resistance or type-2 diabetes mellitus [41–43]. Lowering dietary BCAAs increased energy expenditure and improved insulin sensitivity in animal models [44]. On the contrary, one study showed higher amounts of dietary BCAAs to be associated with lower T2DM risk. [45] Nevertheless, subjects with diabetic concerns were suggested to closely follow up the variation of blood glucose level while taking BCAAs.

Although different changes of sarcopenic parameters were noted between the groups aged < 65 years and ≥ 65years, the interference of the relatively small sample size and diverse distribution of gender cannot be overlooked. Moreover, the decline of sarcopenic parameters after discontinuation was partially compatible in both age groups. These preliminary findings might reflect a potential role of aging in response to BCAA intervention.

Our study had limitations. First, we analyzed only 33 samples with seven dropouts during the discontinuation period and had no randomized placebo group. The results indicate that no statistical difference of basic characteristics was found between the seven dropouts and the other 26 per protocol patients (Supplementary Table 3). Nonetheless, several studies have enrolled less than 30 subjects. For example, the HMB supplementation trial recruiting 16 subjects showed changes in muscle mass [46]. Furthermore, our study met the calculated minimum sample size. In addition to 33 samples, our study had six participants in the open-labeled placebo group. Using 1:2 age and sex matched with the six placebo subjects, the 12 BCAA subjects have showed improved changes in sarcopenic parameters at five weeks (Supplementary Table 4). Both the six subjects without BCAA (Supplementary Table 4) and the 12-week discontinuation evaluation (Table 2) can reflect the placebo effect. Therefore, our findings provided useful evidence of the nutritional intervention for sarcopenia, but whether our findings could be extrapolated to younger or non-sarcopenic groups was uncertain. Second, although we asked the participants to maintain their daily diets and activities as usual during intervention and discontinuation, our study was observational and some may not have compiled. The influence of daily physical activity and dietary food cannot be overlooked in further studies. However, with unchanged lifestyle intervention, our study results would be much closer to real-world interventions and applied in community dwelling populations. Third, we used only two sachets of enriched BCAAs daily. Future studies need more groups with different treatment doses and durations. Fourth, this study was initiated before the announcement of 2019 AWGS [47]. Compared with the new definitions (handgrip, gait speed, SMI) of 2019 AWGS, the enrolled case number remains unchanged. Finally, we did not examine biochemical parameters; thus, we did not examine changes in endocrine and hormone biomarkers.

Supplementation with enriched BCAAs for five weeks may help improve parameters with low values (SMI, gait speed, and grip strength) in pre-sarcopenic and sarcopenic subjects. After 12 weeks discontinuation, these improvements will be obscured, especially SMI and grip strength. However, further investigation is warranted to confirm the optimal types, doses, and durations of treatment and discontinuation effects in clinical practice.

## MATERIALS AND METHODS

### Participants

**Figure 1:**
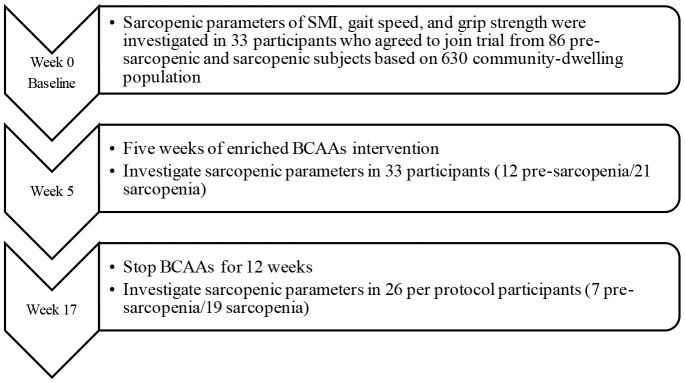
**Flowchart of study participants enrolled from community survey.**

A quasi-experimental single-arm intervention followed by discontinuation study was designed (Figure 1). We surveyed 630 community-dwelling middle-aged and elderly Taiwanese who lived close to Jia-Li Chi Mei Hospital. Based on the power calculation from the Wilcoxon signed-rank test (effect size 0.5, power 0.95, pre- and post-intervention matched pairs; or Takeuchi study [19]), we estimated that at least 29 to 33 participants would satisfy the requirement of statistical analysis. All eligible pre-sarcopenic and sarcopenic recruits were enrolled. Exclusion criteria were uncontrolled hypertension or diabetes, stroke; severe liver or renal disease, gastrointestinal disease, neuromuscular disease, infectious disease, pulmonary disease, endocrine system disease, neurological or acute/advanced psychiatric disease; cancer, a history of seizures, and sensitivity to any study ingredients.

### Assessments

An overview of the interventional study design is shown in Figure 1. At the initial screening, recruits underwent a review of the inclusion and exclusion criteria, medical history, concomitant therapies, daily activity evaluation, and anthropometric and body composition measurements. The short form Mini-Nutritional Assessment (MNA) was used to evaluate nutritional status [48, 49].

Bodyweight and standing height were measured using a medical weight- and height-analyzing scale (Detecto, Webb City, MO, USA); participants were barefoot and dressed in light clothing. Body mass index (BMI) was calculated using this formula: BMI = bodyweight in kg/height in m^2^. Waist circumference (WC) was measured to the nearest mm using a tape measure (Gulick II; Gays Mills, WI, USA) midway between the lateral lower rib margin and the superior anterior iliac crest after a gentle expiration [50]. A single frequency 8-electrode bioelectrical impedance analysis (BIA) device (BC-418; Tanita Corp., Itabashi-ku, Tokyo, Japan) was used to measure body composition, including body fat and skeletal muscle mass (SMM) [51] (estimated using Janssen’s equation (SMI = kg/m^2^) [1, 52–54]. Gait speed was measured by the walking test modified to a 6-meter distance [53]. Low physical performance is predicted when gait speed is < 0.8 m/s [55]. Grip strength (Grip-D [TKK 5401]; Japan) was obtained from three separate 30-s fast-twist tests for one hand for each participant. The maximum value of grip strength was used to assess low-level muscle function based on the corresponding cut points of < 26 kg/m^2^ for men and < 18 kg/m^2^ for women [54, 55]. An SMI < the cut points (7.6 kg/m^2^ for men and 5.67 kg/m^2^for women) was classified as pre-sarcopenia. Sarcopenia was defined as a low SMI and either a low grip strength or a low walking speed by the modified 2014 consensus of the Asian Working Group on Sarcopenia [55].

### BCAA interventions

Eighty-six (13.7%) of 630 participants were pre-sarcopenic or sarcopenic. Twelve pre-sarcopenic and 21 sarcopenic participants were purposively sampled for one sachet enriched BCAAs (Amino VITAL^®^ PRO 3600; Ajinomoto Co., Inc., Tokyo, Japan, 3.6g/sachet) interventions [56] twice daily for 5 weeks. One sachet included leucine 0.54g, isoleucine 0.43g, valine 0.36g, glutamine 0.65g, arginine 0.61g and other amino acids 1.01g. A total of 1.08g leucine per day was supplied for each participant. All participants were asked to habituate themselves to their diet and activity during the study. Participants were contacted by phone every week to discuss compliance, adverse events, and study requirements. The compliance of 5-week BCAAs supplement usage was 97%. After 5 weeks of BCAAs, all BCAA-treated participants discontinued them for the next 12 weeks. Serial sarcopenia-associated tests were done at week 0, week 5, and week 17 for each participant.

### Statistics

All statistical analyses were done using the Statistical Package for the Social Sciences 22 for Windows (IBM Corp., Armonk, NY, USA). Categorical and continuous variables are expressed as percentages or as means ± standard deviation (SD), as indicated. Continuous variables were analyzed using the Wilcoxon Signed Rank non-parametric test. The Mann-Whitney non-parametric test was used to compare dropouts and study completers. The non-parametric Friedman and Wilcoxon Signed Rank test was used to compare the means of variance within three groups and subgroups under the age of 65. Significance was set at p < 0.05 for two-tailed analysis.

### Ethical approval

The study (ClinicalTrials.gov Identifier: NCT03891134) was approved and monitored by the Institutional Review Board of the Chi Mei Medical Center (CMMC10504-J01). Each participant was informed of the purpose of the study, experimental procedures, and potential risks after providing signed written consent.

## Supplementary Material

Supplementary Figure 1

Supplementary Tables
