# Supplementary material for: Effects of enriched branched-chain amino acid supplementation on sarcopenia
Source: Aging (Albany NY). 2020 Jul 26;12(14):15091–103. doi: 10.18632/aging.103576 (PMC7425429; doi:10.18632/aging.103576)
Supplement: Supplementary Figure 1 [file aging-12-103576-s002..pdf]

SUPPLEMENTARY FIGURE

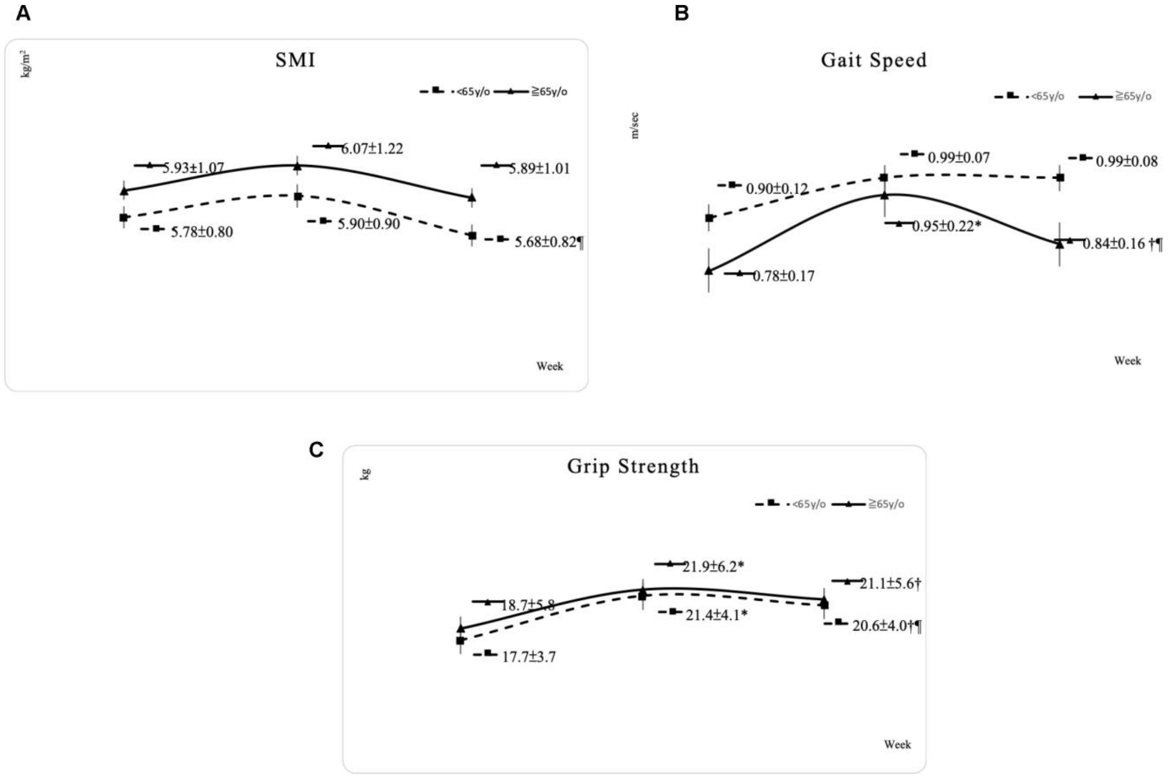

Supplementary Figure 1. Mean (± SD) change from baseline, 5-weeks enriched BCAA intervention period to the 12-week withdrawal period by < 65y/o (n=12, 1 M/11 F) and ≥ 65y/o (n==14, 4 M/10 F) subgroup. (**A**) skeletal muscle mass index (SMI). (**B**) gait speed. (**C**) grip strength. Group differences were analyzed using Non-parametric Friedman & Wilcoxon Signed Rank test. Comparisons between baseline and week 5: * $p < 0.01$. Comparisons between baseline and week 17: † $p < 0.05$. Comparisons between week 5 and week 17: ¶ $p < 0.05$.
