# Supplementary material for: Effects of enriched branched-chain amino acid supplementation on sarcopenia
Source: Aging (Albany NY). 2020 Jul 26;12(14):15091–103. doi: 10.18632/aging.103576 (PMC7425429; doi:10.18632/aging.103576)
Supplement: Supplementary Tables [file aging-12-103576-s001..pdf]

SUPPLEMENTARY TABLES

Supplementary Table 1. Comparison of physical status and performance before and after 5 weeks of enriched branched chain amino acids in sarcopenic participants.

Variable	Baseline	Week 5
Sex	4 M/17 F	
Age (years)	70.9 ± 8.2	
Height (cm)	153.3 ± 5.2	
Mini Nutrition Assessment Short Form score	12.0 ± 1.6	
International physical assessment activity (kcal/week)	5230.1 ± 4534.0	
Body Weight (kg)	50.5 ± 8.5	50.9 ± 9.1*
Body Mass Index (kg/m^2)	21.4 ± 3.1	21.6 ± 3.4*
Body Fat (%)	29.6 ± 5.8	29.1 ± 6.4
Waist Circumference (cm)	80.4 ± 11.6	80.8 ± 12.0
Skeletal Muscle Index (kg/m^2)	5.69 ± 0.90	5.93 ± 1.05**
Gait Speed (m/sec)	0.78 ± 0.18	0.92 ± 0.22**
Grip Strength (kg)	17.9 ± 5.0	20.9 ± 4.9***

Non-parametric Wilcoxon signed rank test: *: $p < 0.05$, **: $p < 0.01$, ***: $p < 0.001$.

Supplementary Table 2. Comparison of physical status and performance before and after 5 weeks of enriched branched chain amino acids in pre-sarcopenic participants.

Variable	Baseline	Week 5
Sex	2 M/10 F	
Age (years)	59.3 ± 9.7	
Height (cm)	155.3 ± 6.3	
Mini Nutrition Assessment Short Form score	12.1 ± 1.4	
International physical assessment activity (kcal/week)	5656.7 ± 5599.9	
Body Weight (kg)	48.3 ± 9.1	48.4 ± 9.6
Body Mass Index (kg/m^2)	19.9 ± 2.71	20.0 ± 2.8
Body Fat (%)	24.6 ± 5.5	24.7 ± 6.1
Waist Circumference(cm)	72.4 ± 11.6	72.8 ± 10.3
Skeletal Muscle Index (kg/m^2)	6.09 ± 0.90	6.09 ± 0.97
Gait Speed (m/sec)	0.88 ± 0.13	0.97 ± 0.09*
Grip Strength (kg)	18.2 ± 4.6	22.3 ± 5.6**

Non-parametric Wilcoxon signed rank test: *: $p < 0.05$, **: $p < 0.01$.

Supplementary Table 3. No statistical difference of sarcopenic parameters between per protocol (PP) participants and dropouts at baseline and after 5 weeks of enriched branched-chain amino acid supplements.

Variable	Baseline		Week 5	
	Dropouts	PP	Dropouts	PP
Sex	1 M/6 F	5 M/21 F	1 M/6 F	5 M/21 F
Age (years)	67.4 ± 11.3	66.4 ± 10.2	67.4 ± 11.3	66.4 ± 10.2
Height (cm)	152.8 ± 4.0	154.3 ± 6.0	152.8 ± 4.0	154.3 ± 6.0
IPAQ (kcal/week)	3442.3 ± 1436.2	5935.4 ± 5964.0	3442.3 ± 1436.2	5935.4 ± 5964.0
Body Weight (kg)	47.0 ± 6.0	50.4 ± 9.2	47.6 ± 6.0	50.7 ± 9.9
Body Mass Index (kg/m^2)	20.2 ± 3.3	21.1 ± 3.0	20.5 ± 3.4	21.2 ± 3.2
Body Fat (%)	26.6 ± 7.0	28.1 ± 6.0	25.9 ± 7.4	27.9 ± 6.5
Waist Circumference (cm)	74.5 ± 10.2	78.3 ± 12.6	75.1 ± 9.9	78.6 ± 12.5
Mini Nutrition Assessment short form score	12.9 ± 1.7	12.8 ± 2.8	13.2 ± 1.0	13.4 ± 1.5
Skeletal Muscle Index (kg/m^2)	5.74 ± 0.87	5.86 ± 0.94	5.97 ± 0.86	5.99 ± 1.06
Gait Speed (m/sec)	0.76 ± 0.20	0.83 ± 0.16	0.85 ± 0.22	0.97 ± 0.17
Grip Strength (kg)	18.3 ± 4.5	18.2 ± 4.9	20.4 ± 4.8	21.7 ± 5.2

IPAQ: international physical assessment activity; Statistical analysis using non-parametric Mann-Whitney U test.

Supplementary Table 4. Comparison of sarcopenic parameters between age and sex matched BCAA intervention and non-intervention group.

Group	Non-intervention			Intervention			
	Baseline	5 weeks	Δ(%)	Baseline	5 weeks	Δ(%)	*P**
(presarcopenia/sarcopenia)	(4/2)			(1/11)			
Sex (M/F)	(1/5)			(1/11)			
Age (years)	60.2±9.7			60.0±6.9			0.88
Body Weight (kg)	53.5±11.5	52.9±10.7	-0.9	49.7±8.3	50.0±9.1	0.4	0.19
Body Mass Index (kg/m^2)	21.0±3.3	20.8±3.0	-0.9	20.5±2.5	20.6±2.8	0.4	0.19
Body fat (%)	26.1±4.9	27.4±6.2	4.6	28.2±5.6	27.8±6.4	-2.0	0.10
Skeletal Muscle Index (kg/m^2)	6.59±1.35	6.20±1.04	-5.1	5.70±0.68	5.80±0.54	2.0	<0.05
Gait Speed (m/sec)	0.92±0.19	0.96±0.17	4.4	0.88±0.13	0.95±0.09	9.0	0.36
Grip Strength (kg)	24.4±7.0	23.5±7.0	-3.8	16.7±4.4	20.2±5.1	24.8	<0.01

*Independent-Sample t test, compared the Δ(%) between two groups.
